# Supplementary material for: Human motion recognition and prediction using loose cloth
Source: Nat Commun. 2026 Jan 20;17:807. doi: 10.1038/s41467-025-67509-7 (PMC12824249; doi:10.1038/s41467-025-67509-7)
Supplement: Supplementary file 1 — Supplementary Information [file 41467_2025_67509_MOESM1_ESM.pdf]

Supplementary Information for

Human Motion Recognition and Prediction using Loose Cloth

Tianchen Shen [1*], Sacha Morris [1], Irene Di Giulio [2], Matthew Howard [1*]

[1]Department of Engineering, King's College London, London, WC2R 2LS, England, UK.
[2]School of Basic & Medical Biosciences, King's College London, London, SE1 1UL, England, UK.

[*]Corresponding author(s). E-mail(s): tianchen.shen@kcl.ac.uk; matthew.j.howard@kcl.ac.uk;

Types of Motion Capture System	Advantages	Disadvantages	Applicable Users
Marker-based Optical	High accuracy and sampling frequency, real-time processing	Uncomfortable, complex setup, and small capture volume within a laboratory environment	Researchers, biomechanical analysts, film/game industry, rehabilitation specialists
Markerless Optical	User-friendly, allows movement in a natural environment	Constrained by environmental requirements, lower precision and real-time capabilities	Game developers, Augmented Reality and Virtual Reality applications, fitness tracking, human-computer interaction researchers
Wearable Sensor-based	Measures movements without an external reference, greater portability	Uncomfortable, complex setup	Sports scientists, physical rehabilitation, exoskeleton and prosthetics researchers
Magnetic	High accuracy, immune to line-of-sight issues	Sensitive to magnetic interference from metal objects, small capture range, cannot track high-speed objects	Medical applications (surgery), human-computer interaction, aerospace and virtual reality simulations
Mechanical	High accuracy and efficient	Discomfort, large size, environmental constraints	Robotics, biomechanics research, exoskeleton control and prosthetics development
**Sensorised Garments (Our approach)**	Comfortable (if loose-fitting), easy to use, unobtrusive, portable, extended use, allows movement in a natural environment	Limited exploration of utilising clothing movement for human motion analysis	Everyday users, people who demand high levels of wearing comfort (e.g., the elderly), healthcare and rehabilitation assessments

Supplementary Table 1: Comparison of different types of motion capture systems.

Supplementary Note 1 Comparison of different types of motion capture systems.

Supplementary Note 2 The extended results cover all levels of prediction task difficulty

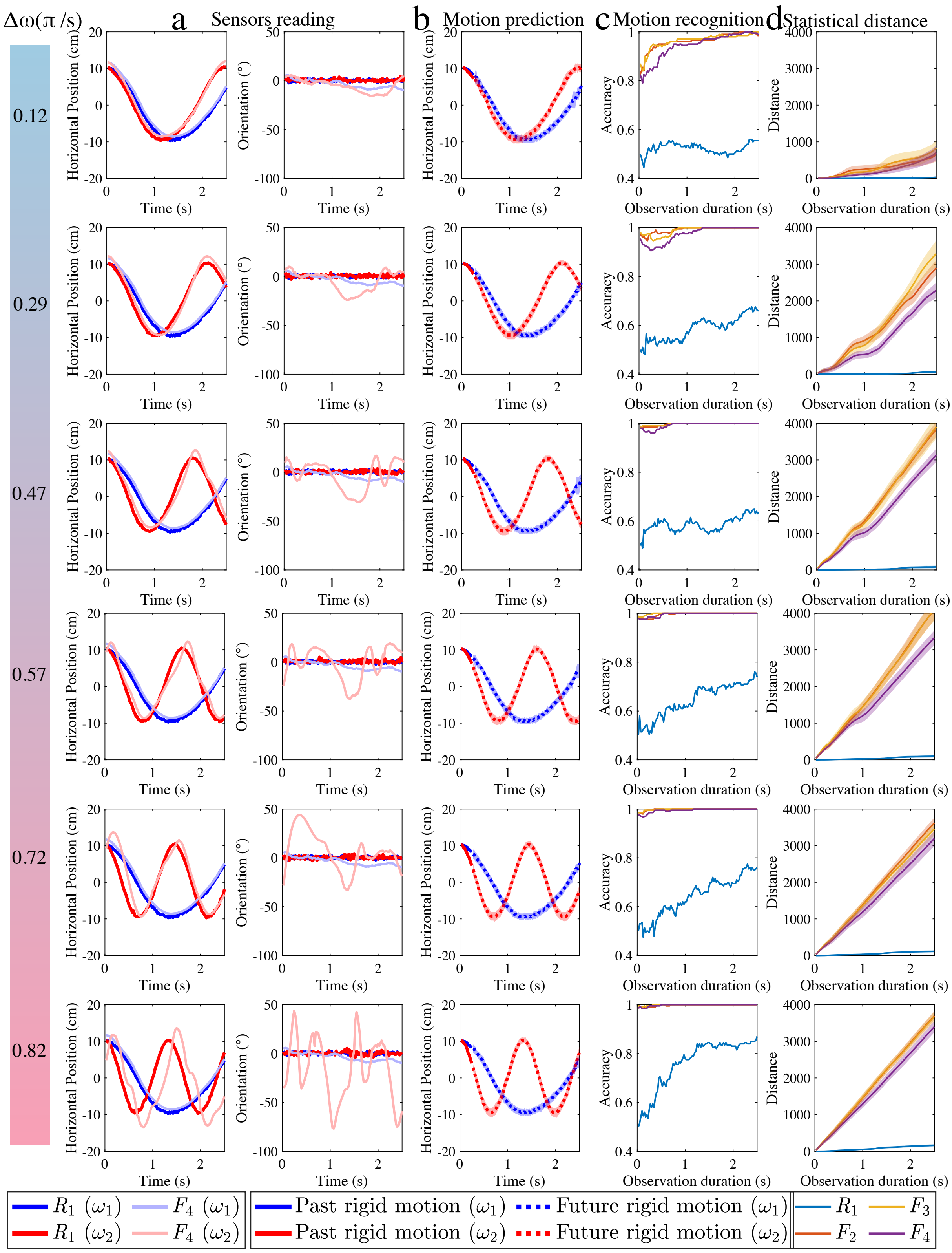

Supplementary Figure 1: (a) The actual horizontal moving trajectory and the yaw orientation of the rigid-attached sensor (R_1 thick and dark line) and the fabric-attached sensor (F_4 thin and light line). (b) The future motion (dashed line and shaded area) is predicted based on the past motion (solid line). The future trajectory shown is the mean value (dashed line) $\pm$ s.d $\times$ 5 (shaded area). (c) The motion recognition accuracy and (d) the statistical distances (mean value solid line; $\pm$ s.d. is shaded area) of each sensor give the past movement from the initial time step to various time steps to 2.5 s.

Supplementary Note 3 Alternative Statistical Modelling Techniques

To verify that the reported effects are not due to some intrinsic bias of the left-to-right hidden Markov model (LR-HMM) model, the analysis has been repeated with other statistical modelling techniques, specifically, temporal convolutional network (TCN) and transformer neural network (TNN)models. These are selected because both architectures are known for their effectiveness when working with time series data. The configuration of these models is as follows.

For the temporal convolutional network, four stacked causal convolution blocks are used with a kernel size of 5 and exponentially increasing dilation rates (1, 2, 4, 8) to capture multi-scale temporal patterns. Each block includes batch normalisation, rectified linear unit (ReLU) activation, and 20% dropout, with residual connections. The three-dimensional rotation of the sensor movement input features is projected onto the 24 channels through a 1×1 convolution. This is followed by a fully connected two-layer classifier: the first layer reduces the 24-dimensional feature vector to 16 dimensions, and the second maps it to the 2 dimensions required for classification, after global average pooling.

The TNN model, designed for small-sample data, projects a three-dimensional rotation of the sensor's movement to a 24-dimensional model space and incorporates positional encoding to preserve temporal ordering. It consists of 2 stacked encoder layers with 2 attention heads each and a compact feedforward network, and uses a classification head that first reduces the 24-dimensional features to 12 dimensions before mapping to the 2 output dimensions, with 10% dropout for regularisation.

Both models are trained using the Adam optimiser (learning rate = 0.001) with early stopping to prevent overfitting, and evaluated via 100 iterations of random subset cross-validation (49 training samples per class, 1 test sample per class per iteration).

Supplementary Figure 2 (a) and (b) show the motion recognition accuracy across four sensors, from the initial time up to various time points within 2.5 s using TCN and TNN. The findings obtained using these two neural network architectures with different structures are consistent with those presented in the manuscript using a LR-HMM (see Fig. 2(e) in the main manuscript).

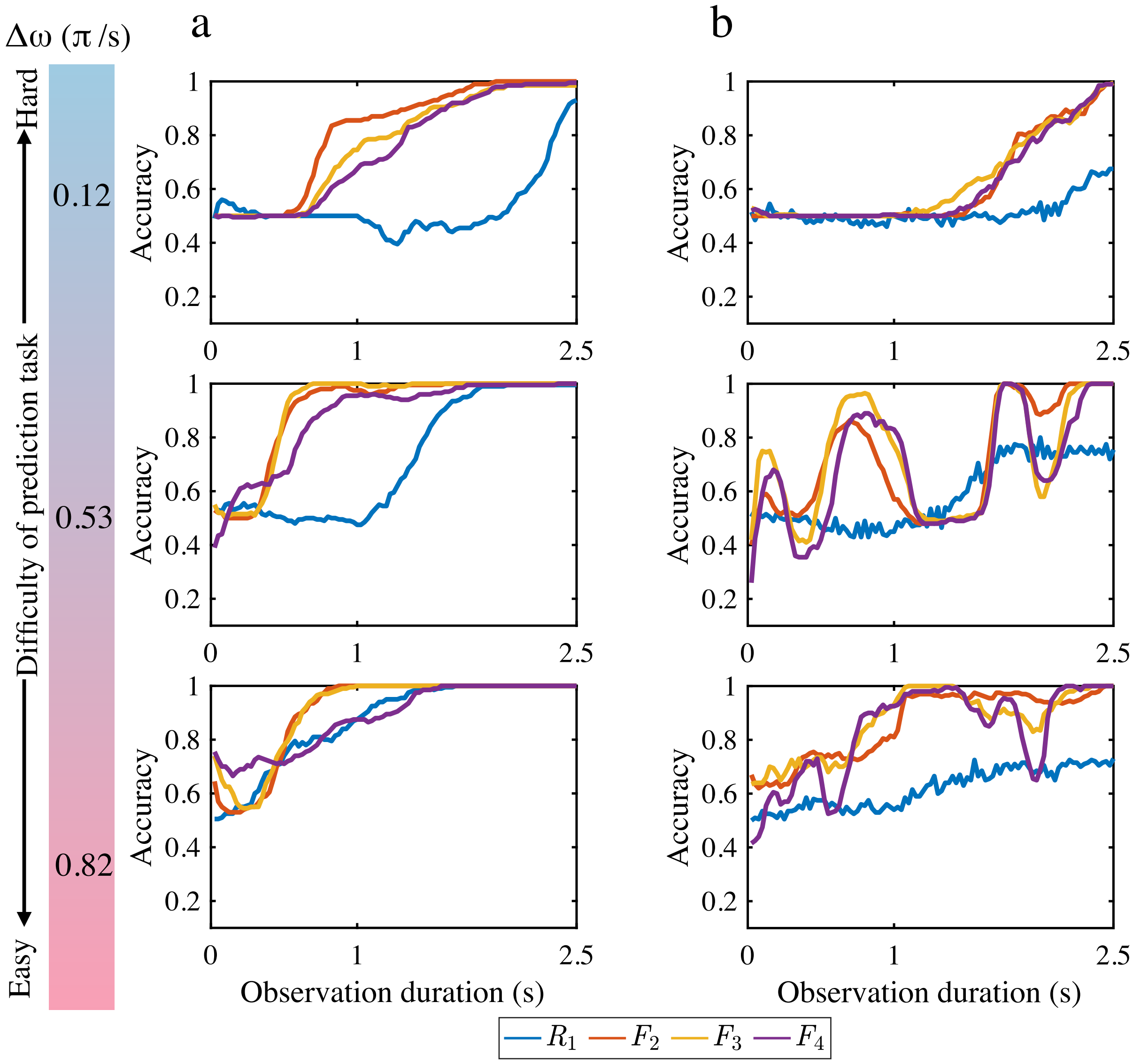

Supplementary Figure 2: The motion recognition accuracy from the initial time to various times up to 2.5 s for all levels of prediction task difficulty using two structures of neural network (a) Temporal Convolutional Network and (b) Transformer.

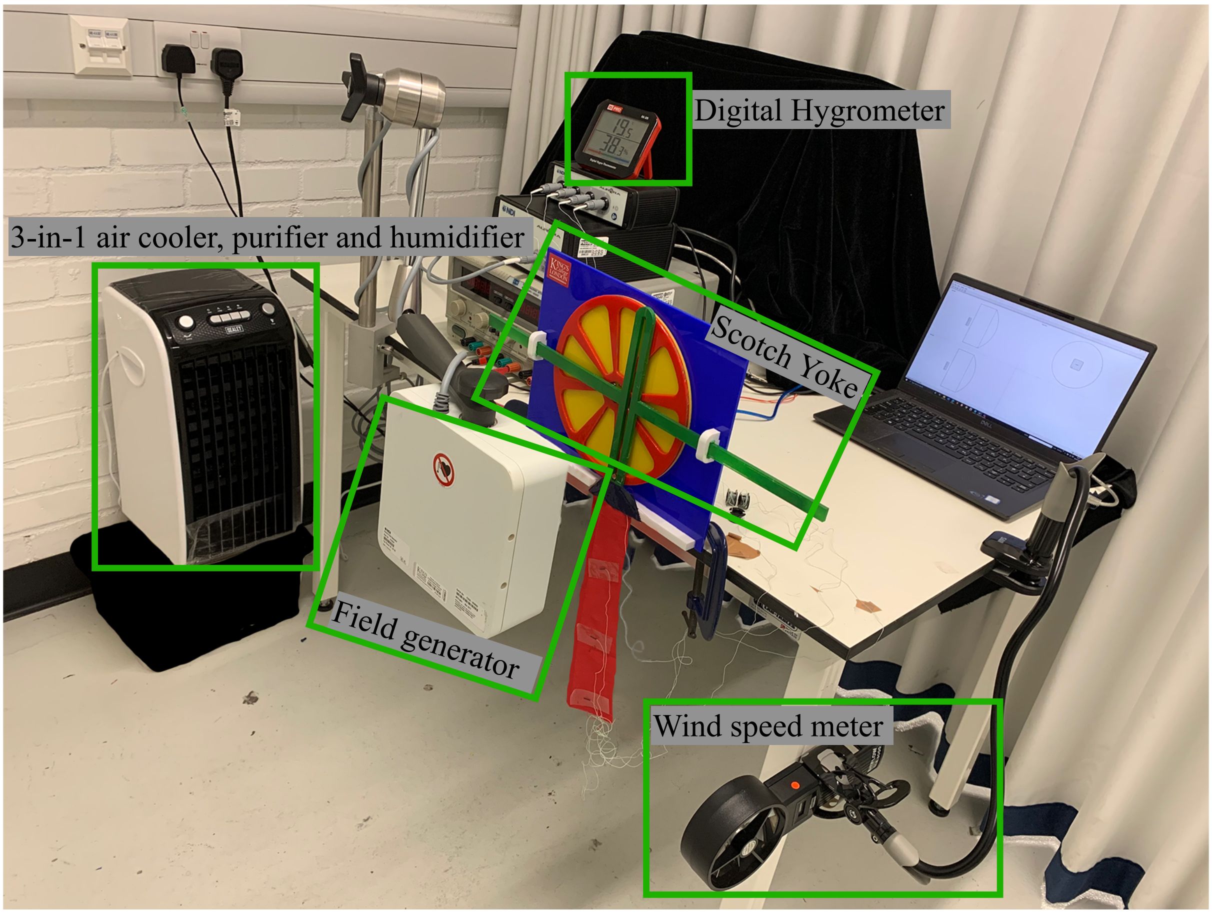

Supplementary Figure 3: Experimental set up for exploring the motion recognition performance of fabric motion under different environments.

Supplementary Note 4 Outdoor environments

Outdoor environmental conditions tend to vary much more than in the indoor settings used for the majority of the experiments reported in this paper. To investigate the potential impact of such factors, the Scotch yoke experiment has been repeated while varying the environmental conditions in a controlled way. Specifically, a multifunctional climate control unit (3-in-1 air cooler, purifier, and humidifier; Sealey UK) is employed to systematically control three key variables: airflow velocity, room temperature, and humidity. The unit is placed 15 cm away from the experimental setup. Supplementary Figure 3 depicts the experimental setup, utilising a wind speed meter to measure airflow velocity and a digital hygrometer to monitor ambient temperature and humidity levels.

To evaluate the impact of environmental conditions on the motion recognition performance of fabric-based sensors, Cohen's d is computed between the recognition accuracy under each test environment and the baseline setup.

Supplementary Note 4.1 Wind speeds

Supplementary Figure 4 shows the motion recognition of the rigid-attached sensor and fabric-attached sensors (*i.e.,* F_2-F_4) for prediction tasks of a variety of difficulties (*i.e.,* $\Delta\omega = \{0.3\pi, 0.47\pi, 0.57\pi, 0.72\pi, 0.82\pi \mathrm{rad\,s^{-1}}\}$) under both still air conditions and controlled airflow velocities (*i.e.,* 0.3 m/s and 0.47 m/s).

Supplementary Table 2 presents Cohen's d values for the motion recognition accuracy of each fabric-attached sensor at different wind speeds (*i.e.,* 0.3 m/s and 0.47 m/s) compared to the no-wind condition. The data indicate that higher wind speeds (0.47 m/s) correspond to larger Cohen's d values, particularly for more challenging prediction tasks ($\Delta\omega = 0.3\pi \mathrm{rad\,s^{-1}}$). Conversely, at lower wind speeds (0.3 m/s), Cohen's d values are smaller, especially for simpler prediction tasks ($\Delta\omega = 0.82\pi \mathrm{rad\,s^{-1}}$).

This observation is further corroborated by the comparison between fabric motion and rigid motion under varying airflow velocities. When the airflow velocity is low (*i.e.,* 0.3 m/s), fabric motion still

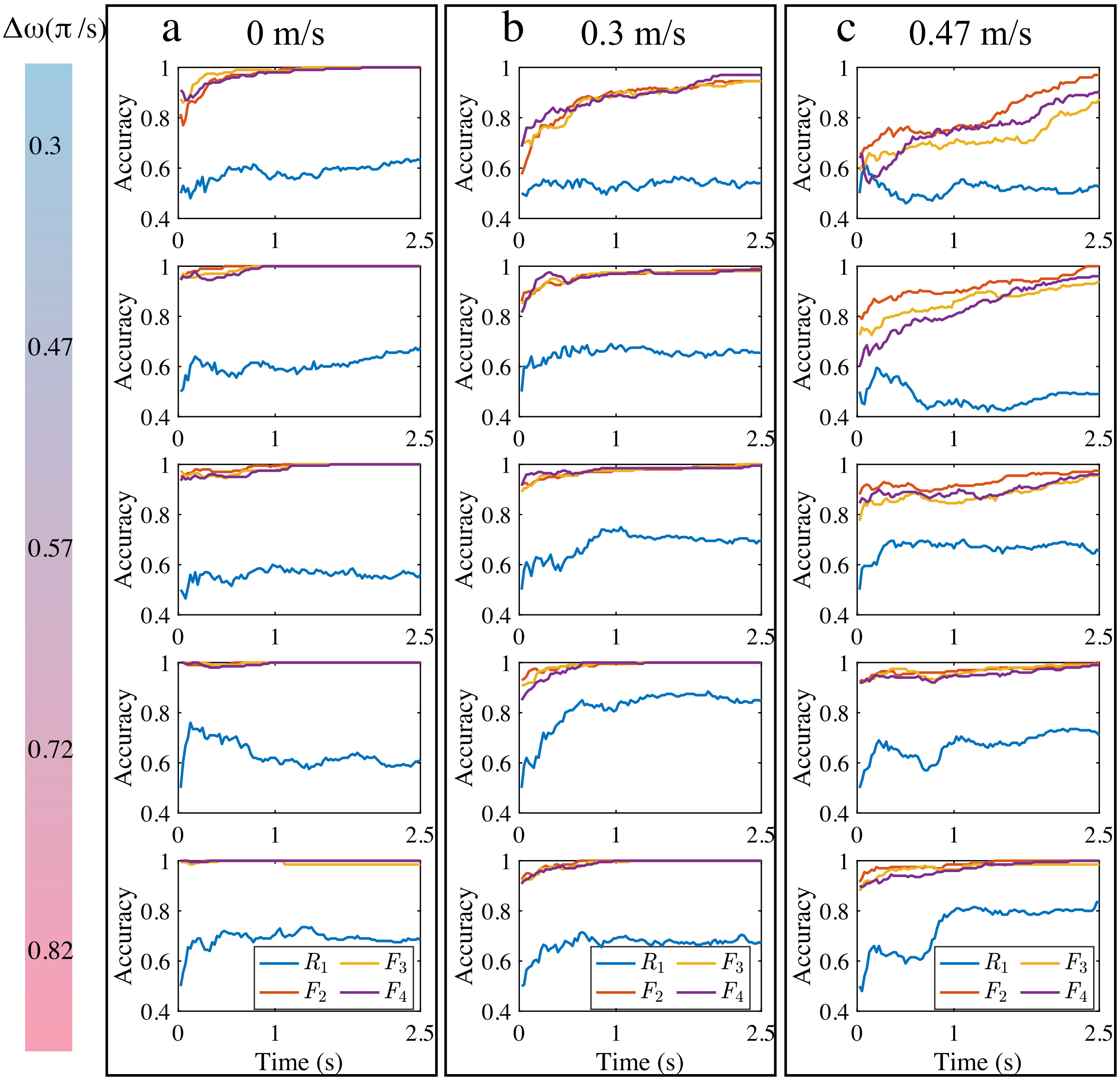

Supplementary Figure 4: Motion recognition accuracy comparison between rigid and fabrics under different wind speeds: (a) 0 m/s (b) 0.3 m/s (c) 0.47 m/s.

exhibits enhanced motion recognition performance compared to rigid motion for challenging prediction tasks, although this advantage is less pronounced than in the absence of external airflow. Conversely, at a higher airflow velocity (*i.e.,* 0.47 m/s), the motion recognition performance of fabric motion is inferior to that of rigid motion, indicating that fabric motion is, as expected, vulnerable to strong winds. For straightforward prediction tasks, there is no substantial difference in the motion recognition performance between rigid and fabric motion. This suggests that while external airflow speed does influence motion recognition from fabric sensors, its impact is negligible in less complex prediction tasks.

Supplementary Note 4.2 Room humidity levels

Supplementary Figure 5 shows the motion recognition of the rigid-attached sensor and fabric-attached sensors (*i.e.,* F_2-F_4) for prediction tasks of a variety of difficulties (*i.e.,* $\Delta\omega = \{0.3\pi, 0.47\pi, 0.57\pi, 0.72\pi, 0.82\pi \mathrm{rad\,s^{-1}}\}$) under varying humidity levels (*i.e.,* 45%, 55% and 65%).

Supplementary Table 3 presents Cohen's d values for the motion recognition accuracy of each fabric-attached sensor at different humidity levels (*i.e.,* 55%, 65%) compared to the baseline condition (*i.e.,* 45%). The consistently small values indicate that humidity variations have no significant effect on the recognition performance of the fabric-based sensors.

$\Delta\omega\pi\mathrm{rad\,s^{-1}}$	F_2		F_3		F_4	
	0.3m/s	0.47m/s	0.3m/s	0.47m/s	0.3m/s	0.47m/s
0.3	1.17	8	1.45	9.5	1.55	8.6
0.47	0.42	2.4	0.65	5.3	-0.09	2.8
0.57	1.3	2.4	1	3.3	1	2.3
0.72	0.2	3	0.02	4	0.3	4
0.82	0.6	2.6	0.7	2.2	0.65	3.2

Supplementary Table 2: Cohen's d values between the motion recognition accuracy of each fabric-attached sensor under different wind speeds (*i.e.,* 0.3 m/s and 0.47 m/s) and no wind setup (*i.e.,* 0 m/s).

$\Delta\omega\pi\mathrm{rad\,s^{-1}}$	F_2		F_3		F_4	
	55%	65%	55%	65%	55%	65%
0.3	-0.3	0.7	-0.57	-0.04	-0.19	0.8
0.47	0.18	0.19	0.16	0.18	-0.5	-0.3
0.57	0.2	-0.3	0.02	-0.3	-0.6	-0.9
0.72	0.2	0.57	-2	-1	-0.3	-0.2
0.82	0.4	0.6	0.3	0.6	-0.3	0.4

Supplementary Table 3: Cohen's d values between the motion recognition accuracy of each fabric-attached sensor under different humidity levels (*i.e.,* 55%, 65%) and the original setup (*i.e.,* 45%).

$\Delta\omega\pi\mathrm{rad\,s^{-1}}$	F_2		F_3		F_4	
	$15°C$	$25°C$	$15°C$	$25°C$	$15°C$	$25°C$
0.3	-0.42	-0.4	-0.4	0.3	0.33	0.4
0.47	-0.17	-0.8	-0.6	-0.9	-1	-0.83
0.57	0.56	-0.47	0.12	-0.74	-0.38	-0.6
0.72	-0.28	-0.7	-0.57	-0.52	-0.88	-0.1
0.82	0.67	0.01	0.02	0.59	0.17	0.4

Supplementary Table 4: Cohen's d values between the motion recognition accuracy of each fabric-attached sensor under different room temperatures (*i.e.,* 15, 25 degrees) and the original setup (*i.e.,* 20 degrees).

Supplementary Note 4.3 Room temperatures

Supplementary Figure 6 shows the motion recognition of the rigid-attached sensor and fabric-attached sensors (*i.e.,* F_2-F_4) for prediction tasks of a variety of difficulties (*i.e.,* $\Delta\omega = \{0.3\pi, 0.47\pi, 0.57\pi, 0.72\pi, 0.82\pi\mathrm{rad\,s^{-1}}\}$) under varying room temperatures (*i.e.,* 15, 20 and 25 degree).

Supplementary Table 4 shows Cohen's d values at different temperatures (*i.e.,* 15, 25 degrees) versus the baseline (*i.e.,* 20 degrees). Here too, the small values demonstrate that temperature variations do not significantly affect the recognition performance of the sensors.

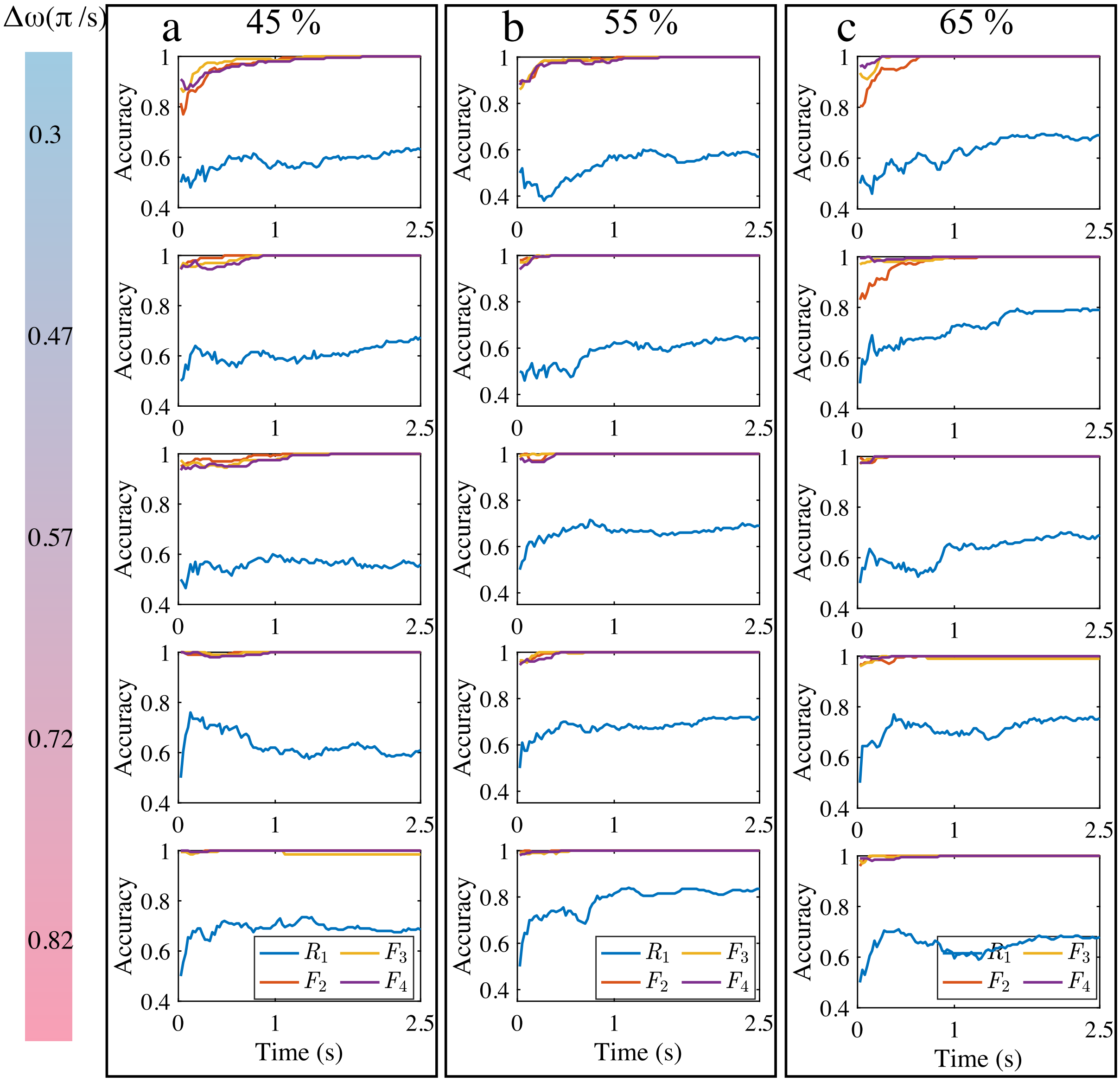

Supplementary Figure 5: Motion recognition accuracy comparison between rigid and fabrics under humidity levels: (a) 45% (b) 55% (c) 65%.

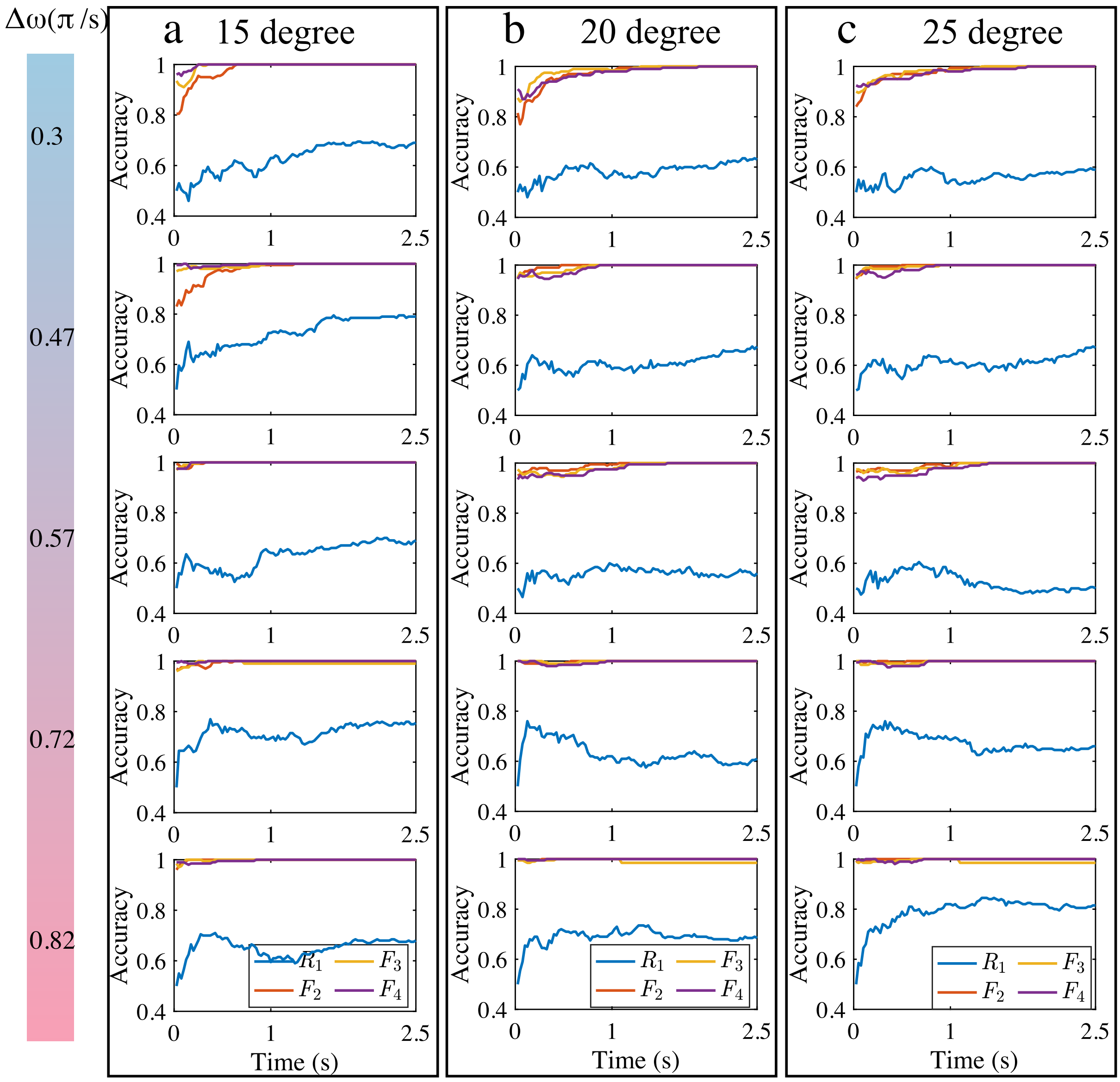

Supplementary Figure 6: Motion recognition accuracy comparison between rigid and fabrics under room temperatures: (a) 15 (b) 20 (c) 25 degrees.

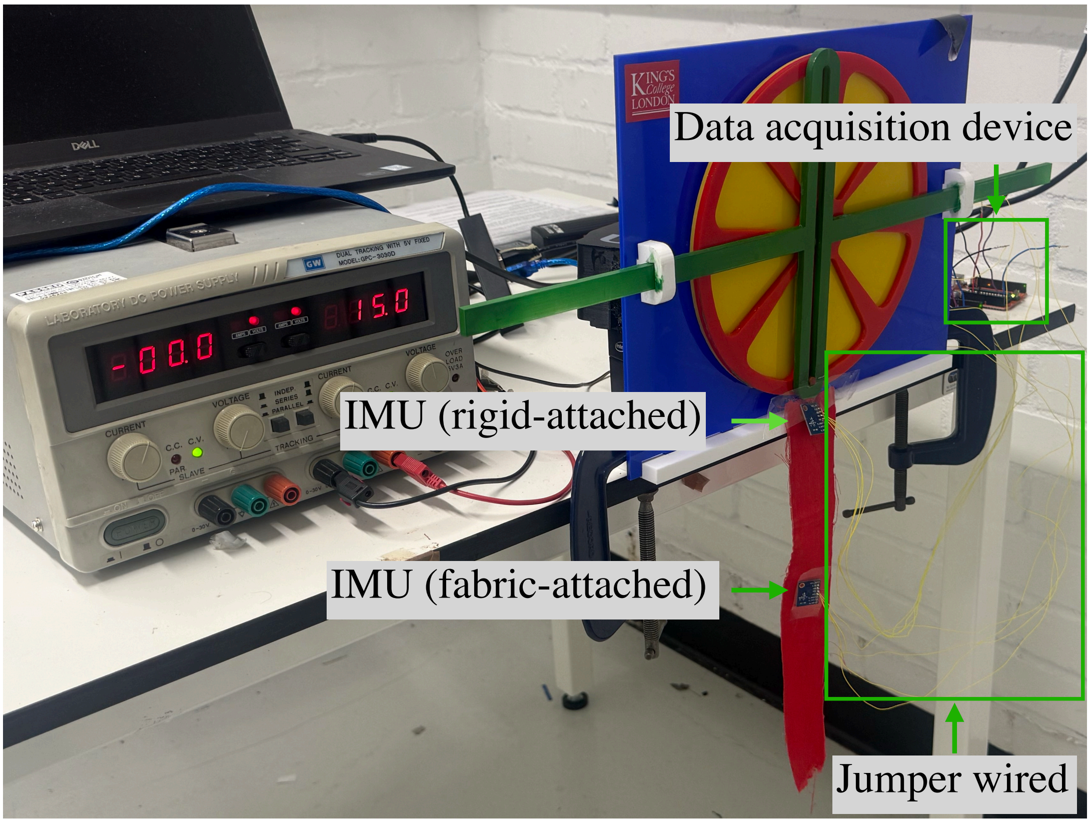

Supplementary Figure 7: The configuration of the experimental setup used to replace the Aurora sensors with IMUs.

Supplementary Note 5 Validation with IMUs

To examine whether inertial measurement units (IMUs) can support the conclusions presented in this paper, the scotch yoke experiment was repeated with the Aurora 6 degree-of-freedom sensors replaced by IMUs. One ADXL335 sensor (Analog Devices, Inc., USA) was attached to the yoke to capture rigid-body motion, and a second sensor was affixed to the fabric. Note that the presence of the IMU (large and heavier than the Aurora sensors) and jumper wires (used to connect the sensor to the data acquisition device) may influence fabric motion. To minimise this effect, only one sensor was attached to the fabric F, as opposed to the three Aurora sensors used in the main experiments. Moreover, the IMU does not directly provide positional or orientation data, which must be computed through integration (that is prone to noise and drift), so motion recognition performance for both rigid and fabric motion was evaluated using three-axis acceleration data from the sensors. The experimental setup of it is shown in Supplementary figure 7.

The motion recognition accuracy of the fabric-attached sensor ranges from 99% to 100%, whereas that of the rigid-attached sensor ranges from 69% to 84% for the prediction task (*i.e.*, $\Delta\omega = 0.3\pi \mathrm{rad\,s}^{-1}$) from time $t = 0.5\,\mathrm{s}$ up to $t = 2.5\,\mathrm{s}$. This demonstrates that the accuracy achieved using fabric-based motion remains significantly higher than that obtained using rigid motion.

Supplementary Note 6 The extended the moving patterns of the robot cover all levels of prediction task difficulty

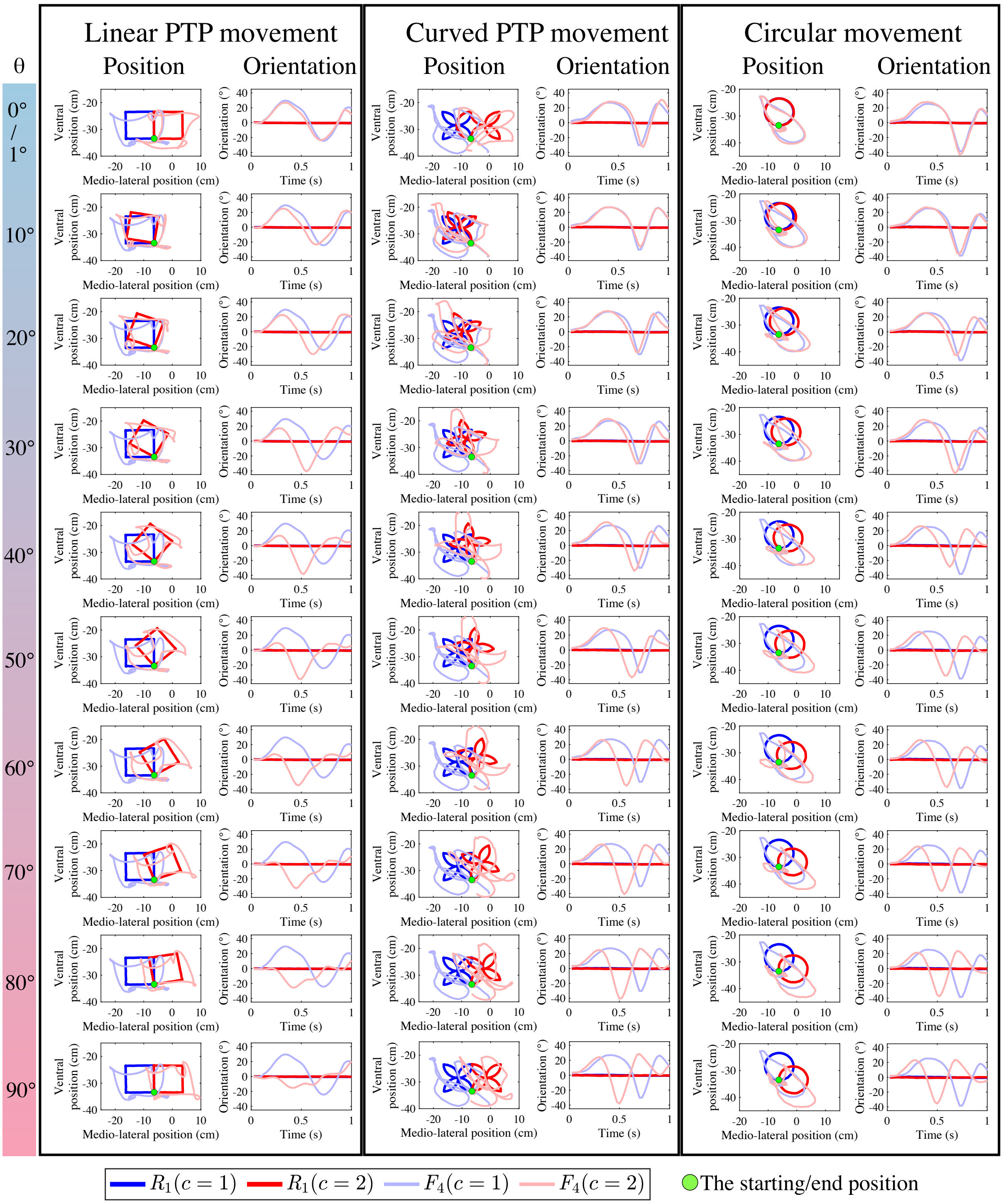

Supplementary Figure 8: The robot arm follows three types of predefined trajectories (front view), displaying the actual movement trajectory and the yaw orientation of both the rigid R_1 and fabric-attached sensors F_4 when the end effector of the robot arm moves at $\dot{r} = 2.25\text{cm}\,\text{s}^{-1}$. The moving pattern of sensor R_1 is the same for all moving velocities of the robot arm. The movement patterns captured by sensor F_4 show variability.

Supplementary Note 7 The extended results for all levels of prediction task difficulty when the robot arm follows linear point-to-point (PTP) movement and moves at 2.25 $\mathrm{cm\,s^{-1}}$

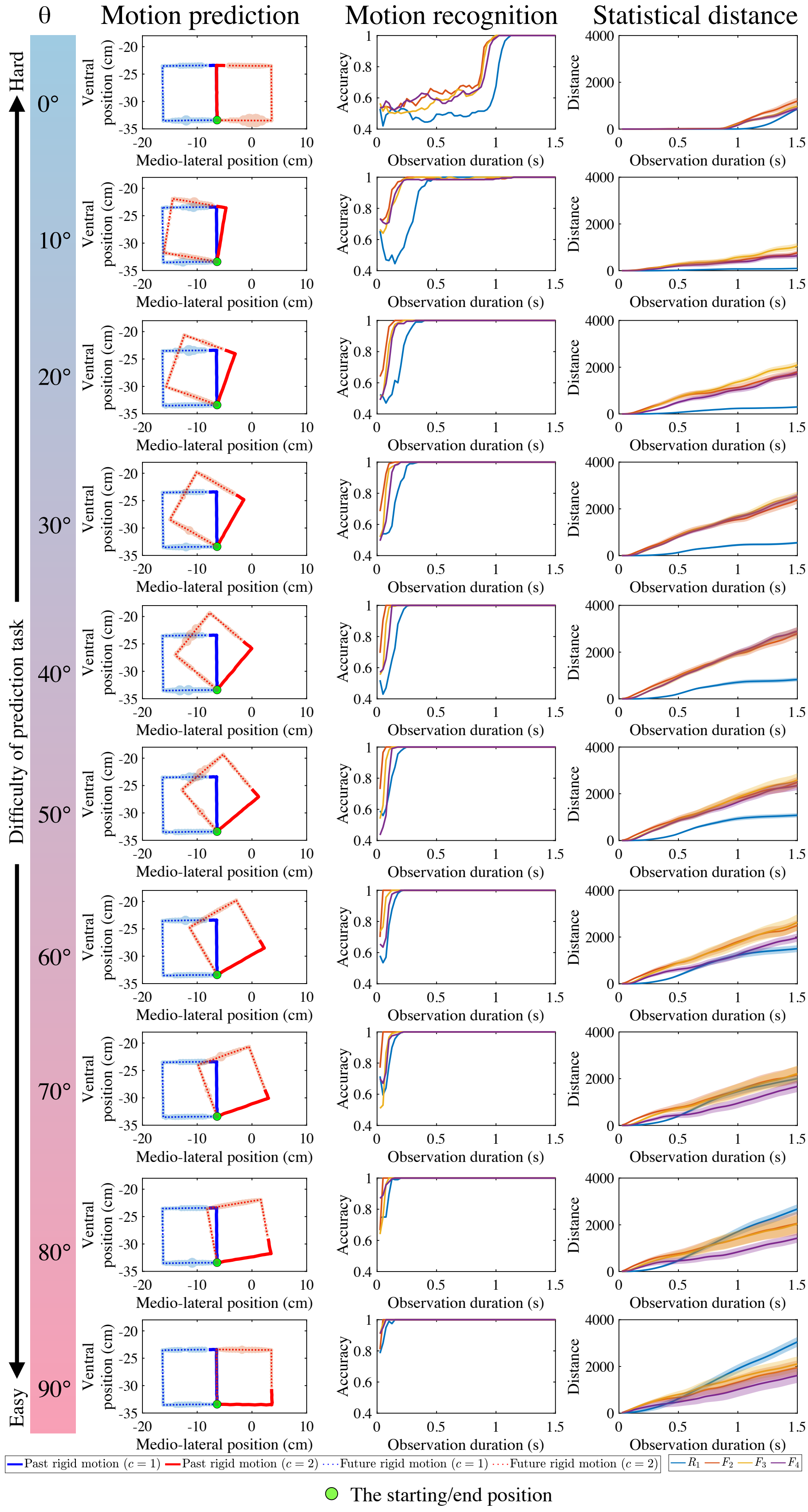

Supplementary Figure 9: The future motion (dashed line and shaded area) is predicted based on the past motion (solid line). The future trajectory shown is the mean value (dashed line) $\pm$ s.d $\times$ 5 (shaded area). The motion recognition accuracy and the statistical distances (mean value solid line; $\pm$ s.d. is shaded area) of each sensor give the past movement from the initial time step to various time steps to 1.5 s.

Supplementary Note 8 The extended results for all levels of prediction task difficulty when the robot arm follows curved PTP movement and moves at $2.25\,\mathrm{cm\,s^{-1}}$

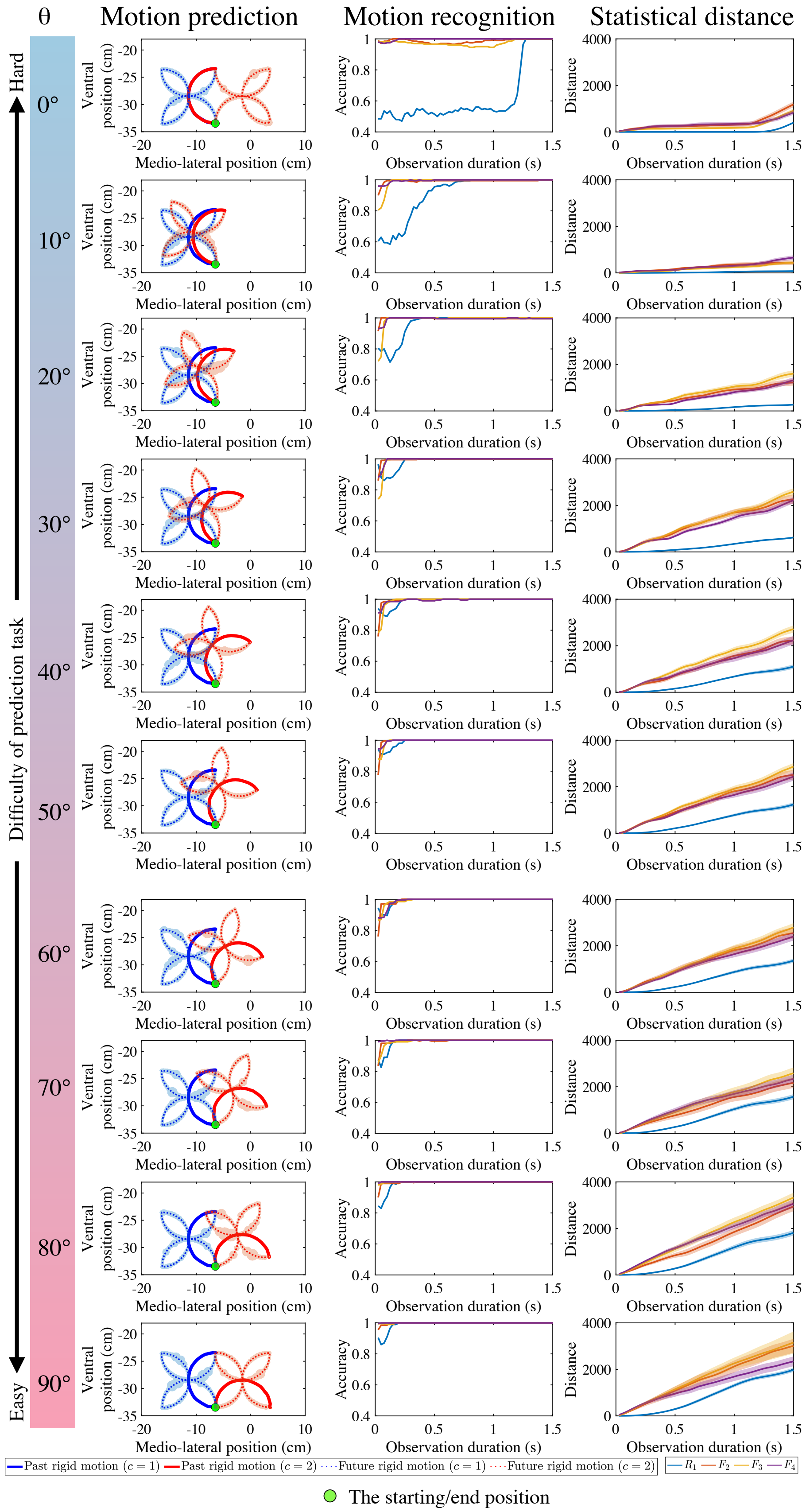

Supplementary Figure 10: The future motion (dashed line and shaded area) is predicted based on the past motion (solid line). The future trajectory shown is the mean value (dashed line) $\pm$ s.d $\times$ 5 (shaded area). The motion recognition accuracy and the statistical distances (mean value solid line; $\pm$ s.d. is shaded area) of each sensor give the past movement from the initial time step to various time steps to 1.5 s.

Supplementary Note 9 The extended results for all levels of prediction task difficulty when the robot arm follows circular movement and moves at 2.25$\mathrm{cm\,s^{-1}}$

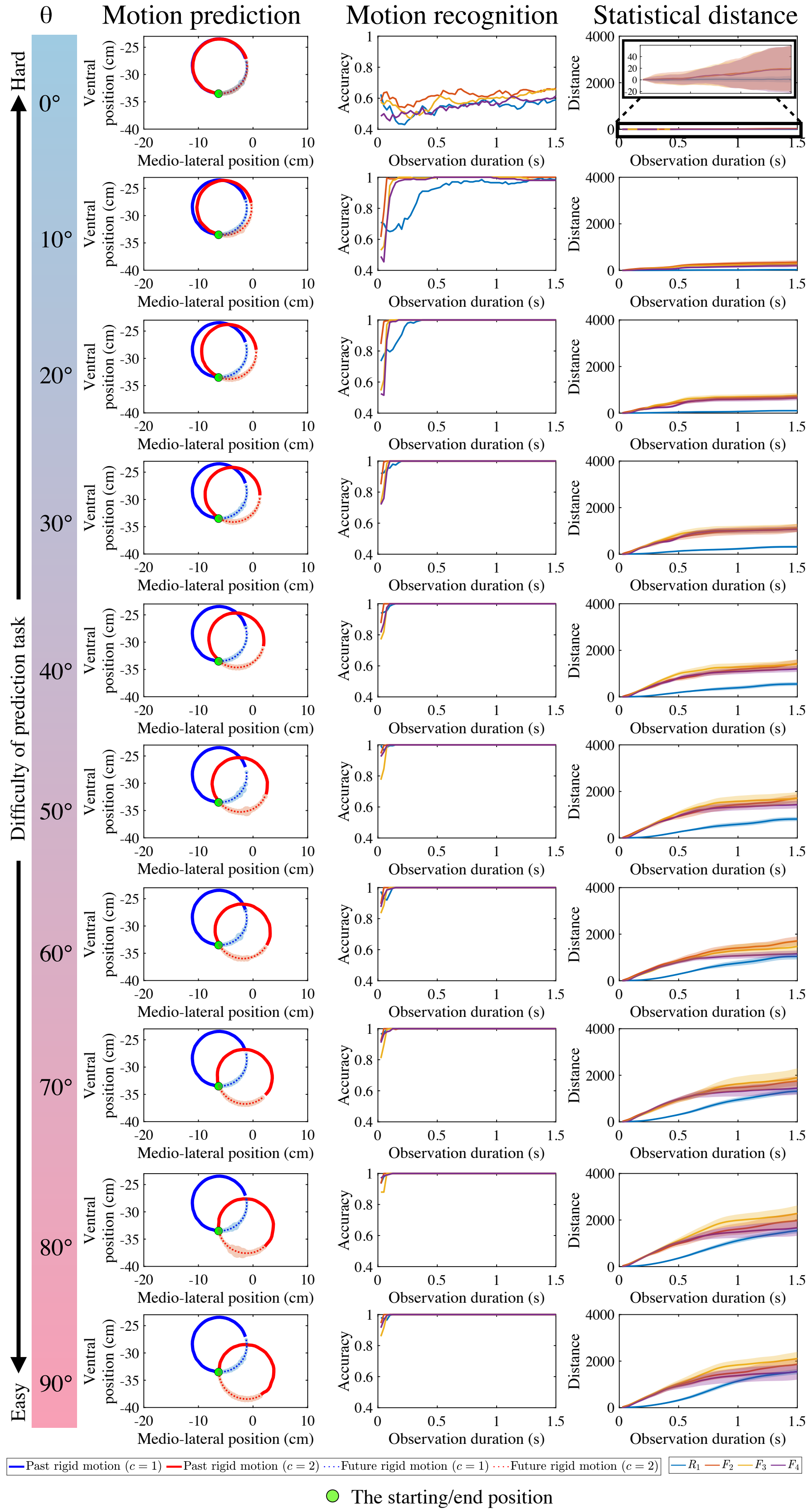

Supplementary Figure 11: The future motion (dashed line and shaded area) is predicted based on the past motion (solid line). The future trajectory shown is the mean value (dashed line) $\pm$ s.d $\times$ 5 (shaded area). The motion recognition accuracy and the statistical distances (mean value solid line; $\pm$ s.d. is shaded area) of each sensor give the past movement from the initial time step to various time steps to 1.5 s.

Supplementary Note 10 Extended Results include motion recognition accuracies for different task difficulties and arm speeds

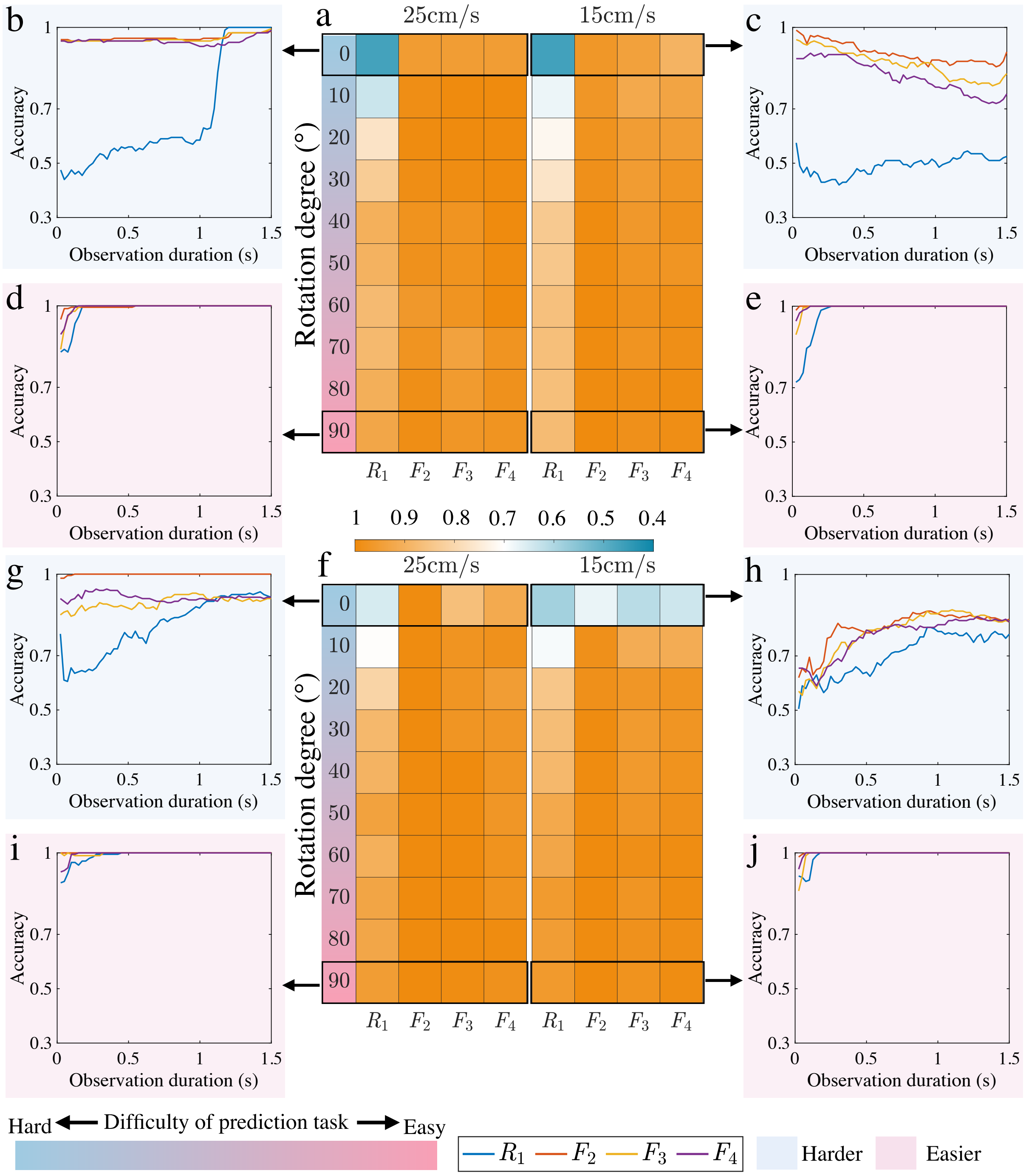

Supplementary Figure 12: (a) Heatmaps show mean motion recognition accuracies from the initial time to 0.25 s across various rotation degrees in the context of curved PTP and (b) In the context of curved PTP movement, the motion recognition accuracy of each sensor give the past movement from the initial time step to various time steps to 1.5 s when the robot arm is moving at (c) 25cm s^{-1} and (d) 15cm s^{-1} for the harder prediction tasks (*i.e.*, $\Delta\theta = 0°$). The motion recognition accuracy of each sensor give the past movement from the initial time step to various time steps to 1.5 s when the robot arm is moving at (e) 25cm s^{-1} and (f) 15cm s^{-1} for the easier prediction tasks (*i.e.*, $\Delta\theta = 90°$). In the context of circular movement, the motion recognition accuracy of each sensor give the past movement from the initial time step to various time steps to 1.5 s when the robot arm is moving at (g) 25cm s^{-1} and (h) 15cm s^{-1} for the harder prediction tasks (*i.e.*, $\Delta\theta = 0°$). The motion recognition accuracy of each sensor give the past movement from the initial time step to various time steps to 1.5 s when the robot arm is moving at (i) 25cm s^{-1} and (j) 15cm s^{-1} for the easier prediction tasks (*i.e.*, $\Delta\theta = 90°$).

Supplementary Note 11 Extended Description of human reaching motion

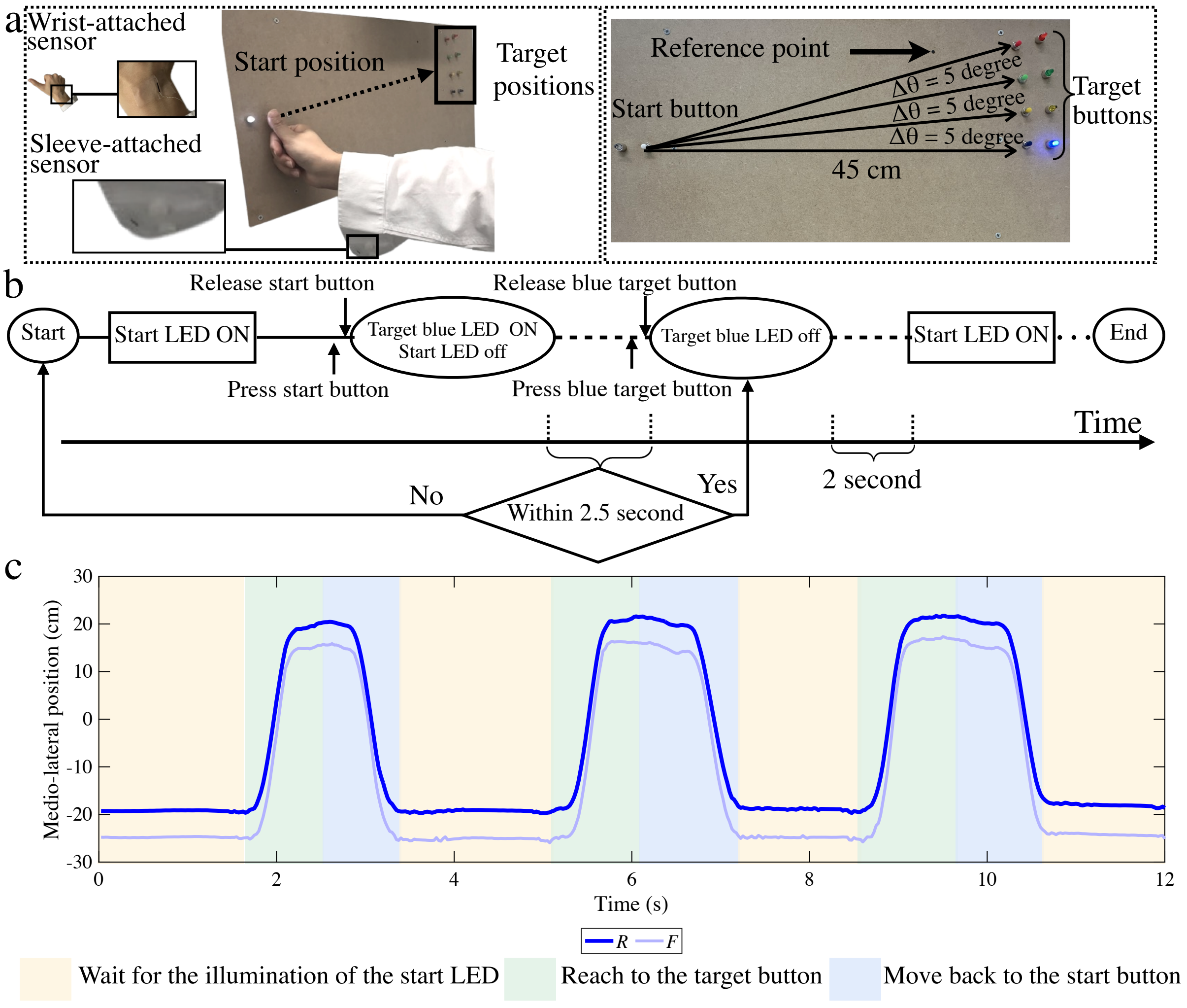

Supplementary Figure 13: (a) The sensors placments and the position diagram of the buttons and LED lights. (b) An example of the flow chart for one human reaching movement starting the LED lighting and pressing the blue target button. (c) The actual sensors reading (*i.e.*, R, F) in the partly data collection process.

Supplementary Note 12 Probabilistic Model of Fabric Motion

The effect of fabric motion on the data $\mathbf{X}^f$ is subject to a high degree of uncertainty, arising from challenges in estimating the fabric's physical properties and the resultant movement complexity. To deal with this, it is proposed to model the data generation process through stochastic methods.

Specifically, the orientation y^f of (*i.e.*, $\mathcal{M} = 1$) the point p^f_y on the fabric at any given time is modelled as the stochastic process consisting of the corresponding orientation y^r (*i.e.*, $\mathcal{M} = 1$) of a point p^r_y on the rigid body plus a random offset $\boldsymbol{\delta}$ introduced by the fabric motion. In the univariate case, this can be written as

$$X^f = X^r + \Delta, \tag{1}$$

where $y^f(t) \sim X^f$, $y^r(t) \sim X^r$ and $\delta(t) \sim \Delta$.

The key to determining whether the fabric movement is beneficial to motion recognition is to understand the effect of Δ on the statistical distance between movement classes. A condition for improved classification performance is a greater statistical distance in data distribution between movement classes in $\mathbf{X}^f$ compared with $\mathbf{X}^r$, *i.e.*,

$$D(X^f_{c=1}, X^f_{c=2}) > D(X^r_{c=1}, X^r_{c=2}), \tag{2}$$

where $X^f_{c=1}$ is the distribution of fabric data for movement class $c = 1$, $X^f_{c=2}$ is the same for movement class $c = 2$, $X^r_{c=1}$ and $X^r_{c=2}$ are the equivalent distributions for rigid data and $D(\cdot, \cdot)$ is a suitable metric in probability space. For the latter, several choices are available (*e.g.*, Kullback-Leibler divergence (KLD)).

Supplementary Note 12.1 Example: Oscillatory Motion

Consider the problem of movement analysis for a one-dimensional, scotch yoke mechanism using data from a sensor mounted on an inextensible piece of fabric attached to the mechanism (see Supplementary Figure 14(a)). Here, a motion recognition task might involve classifying a set of movements of the rigid body (*e.g.*, those with different frequencies) from raw orientation data.

The displacement in orientation data Δ between rigid and fabric motion collected at various frequencies of the Scotch Yoke could be computed using equation 1. The Jarque-Bera test, at the 1% significance level, is employed to determine if the data Δ follows a Gaussian distribution [1].

Frequency (π/s)	F_2	F_3	F_4
0.63	45	50	48
0.75	49	50	50
0.92	50	50	50
1.1	50	50	44
1.2	50	50	44
1.35	50	50	46
1.45	35	49	47

Supplementary Table 5: The number of trajectories passes the Jarque-Bera test (The total number of trajectories is 50).

As can be seen from Supplementary Table 5, the probability density function (PDF) of most trajectories is a Gaussian distribution. This is followed by:

$$\Delta \sim \mathcal{N}(\mu, \sigma^2). \tag{3}$$

Therefore, the variance σ^2 for each frequency of the Scotch Yoke movement can be estimated using maximum likelihood estimation (MLE) [2]. Supplementary Figure 14(b) shows the estimated σ^2 for each sensor as the scotch yoke operates across a range of frequencies. It illustrates that the value of σ^2 increases with the frequency for each sensor, indicating a rise in the uncertainty of the orientation measurements. Following this evidence, a probabilistic model based on the scotch yoke is proposed. Supplementary Figure 14(c) and (d) exhibit an illustrative example of rigid and fabric movements, along with their respective PDF in a yaw orientation, when the scotch yoke operates at varying frequencies.

Supplementary Note 12.2 Statistical Analysis

Based on equation (2), the statistical distance between two movements for rigid and fabric motion can be estimated as follows.

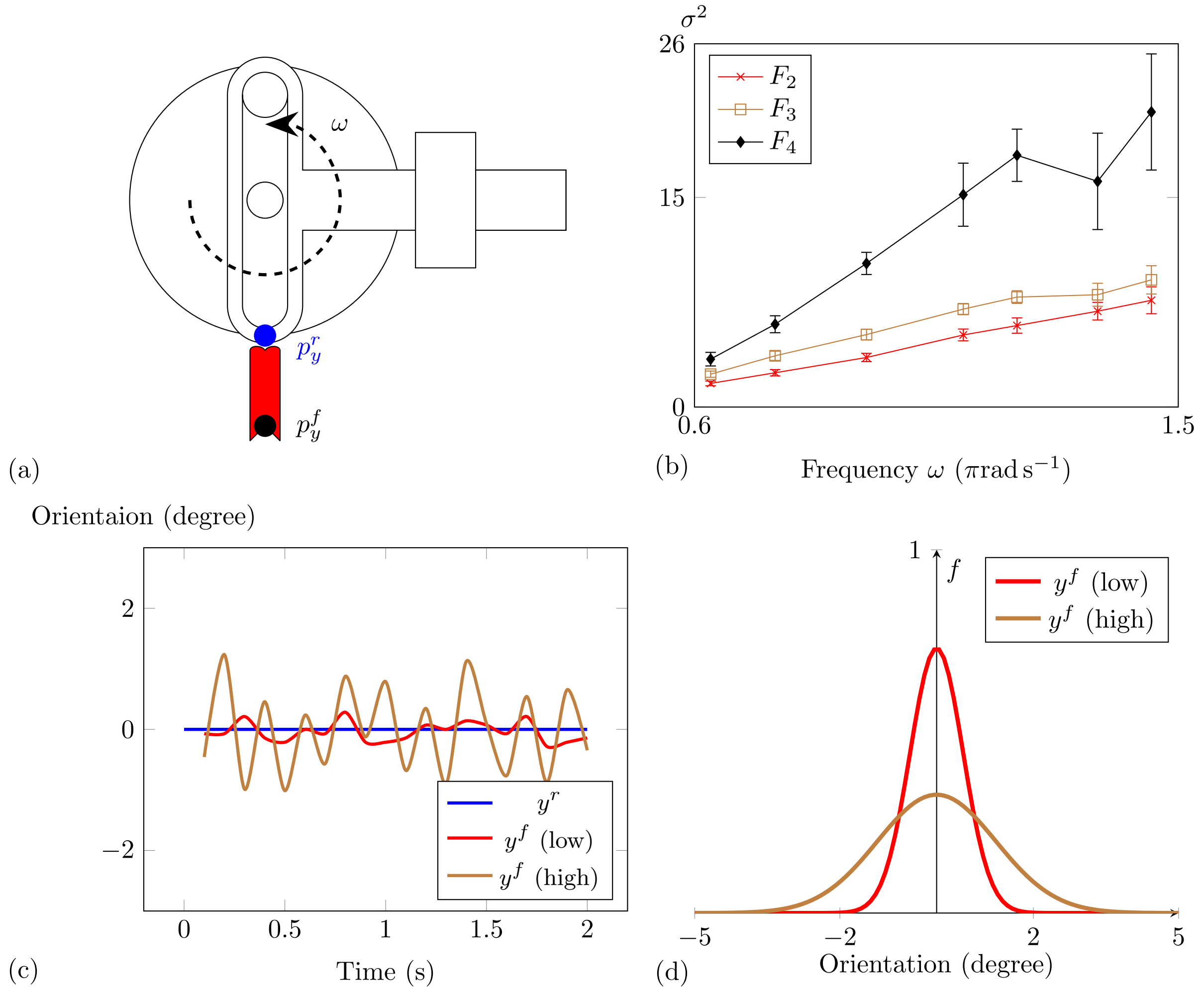

Supplementary Figure 14: (a) A scotch yoke mechanism with a piece of fabric (red) attached. (b) The estimated derivation σ^2 at each frequency for each sensor. (c) An example of rigid and fabric movement. (d) The PDF of fabric movement in orientation when the Scotch yoke operates at low and high frequencies.

The orientation of rigid movement is a constant at all different frequencies. Therefore, it is apparent that the orientation of rigid movement is *independent of* ω, meaning that if the motion recognition task is to classify movements of different frequencies (*e.g.*, ω_1 and ω_2), *the data distribution cannot increase the statistical distance to discriminate between classes, i.e.,* $D_{KL}(X^r_{\omega_1}, X^r_{\omega_2}) = 0$.

However, the dependency of the PDF of X^f on ω is apparent, leading to a statistical distance between movements of different frequencies. The KLD between fabric motion with different frequencies is shown below: [3]

$$D_{KL}(X^f_{\omega_1} \parallel X^f_{\omega_2}) = \log\left(\frac{\sigma_{\omega_2}}{\sigma_{\omega_1}}\right) + \frac{\sigma^2_{\omega_1}}{2\sigma^2_{\omega_2}} - \frac{1}{2} \tag{4}$$

As $D_{KL}(X^f_{\omega_1} \parallel X^f_{\omega_2}) > 0$, the condition (2) is met, suggesting that motion recognition based on the motion of the fabric will lead to higher classification performance. Moreover, $D_{KL}(X^f_{\omega_1} \parallel X^f_{\omega_2}) > 0$ increases monotonically with $\omega_2 - \omega_1$, as can be seen by examining its derivative.

To determine the behaviour of this expression as σ_{ω_2} varies, the derivative with respect to σ_{ω_2} is calculated:

$$\frac{\partial}{\partial\sigma_{\omega_2}} D_{KL}(X^f_{\omega_1} \parallel X^f_{\omega_2}) = \frac{\partial}{\partial\sigma_{\omega_2}}\left(\log\left(\frac{\sigma_{\omega_2}}{\sigma_{\omega_1}}\right) + \frac{\sigma^2_{\omega_1}}{2\sigma^2_{\omega_2}} - \frac{1}{2}\right) = \frac{\sigma^2_{\omega_2} - \sigma^2_{\omega_1}}{\sigma^3_{\omega_2}} \tag{5}$$

Given that $\sigma^3_{\omega_2}$ is always positive, the sign of the derivative depends on the numerator $\sigma^2_{\omega_2} - \sigma^2_{\omega_1}$. If $\sigma_{\omega_2} > \sigma_{\omega_1}$, the numerator, and consequently the derivative, is positive. This indicates that $D_{KL}(X^f_{\omega_1} \parallel X^f_{\omega_2})$ increases as σ_{ω_2} increases, affirming that the KLD indeed expands as the difference $\sigma_{\omega_2} - \sigma_{\omega_1}$ grows. This

suggests that the greater difference in frequency between two movements of the scotch yoke, the greater the statistical distance.

Supplementary Note 13 Computational Complexity analysis

Virtual reality or human-robot collaboration requires a short response time [4]. The following analysis of the computational complexity during the deployment stage with the proposed human motion recognition and prediction framework.

In this approach, during the deployment stage, the computational complexity mainly depends on the number of hidden states $\mathcal{N}$, the dimensionality of the data $\mathcal{M}$, and the number of time steps v. The trajectory from the initial time to 0.25 s (*i.e.,* $v = 10$) is used as an example since all sensors can achieve satisfactory motion recognition accuracy. The following outlines the complexity of LR-HMMs during the deployment stage:

Supplementary Note 13.1 Motion recognition

The first step is motion recognition, which is performed using the forward algorithm. The complexity of this algorithm is [5]

$$O(\mathcal{N}^2 v \mathcal{M}). \tag{6}$$

In the experiments reported in the manuscript, the hardware configuration consists of a Intel Core i7-8700 @3.20GHz ×12 CPU with 16GB memory, running MATLAB 2022b, for which the processing time is approximately 0.0035 s.

Supplementary Note 13.2 Motion prediction

The next step in the process is motion prediction, which relies on the Viterbi algorithm. The Viterbi algorithm is used to find the most likely sequence of hidden states given the observed trajectory. The computational complexity of the Viterbi algorithm is similar to that of the forward algorithm, as it also involves computing probabilities over all hidden states at each time step. However, in this case, we need to perform an additional operation to keep track of the most likely path. The complexity of this algorithm is [5]

$$O(\mathcal{N}^2 \mathcal{V} \mathcal{M}). \tag{7}$$

The processing time is approximately 0.012 s.

Our LR-HMM implementation therefore has 15.5 ms total processing time (3.5 ms for recognition + 12 ms for prediction). This meets the stringent latency requirements of virtual reality systems, where smaller than 20 ms response times are critical for maintaining immersion and preventing motion sickness [6]. For robotic systems that require high-precision collaboration, the delay time should typically be less than 50 ms to ensure precise synchronisation of movements and reduce system reaction time [7].

References

[1] Thorsten Thadewald and Herbert Büning. Jarque–Bera test and its competitors for testing normality–a power comparison. *Journal of applied statistics*, 34(1):87–105, 2007.

[2] Jian-Xin Pan, Kai-Tai Fang, Jian-Xin Pan, and Kai-Tai Fang. Maximum likelihood estimation. *Growth curve models and statistical diagnostics*, pages 77–158, 2002.

[3] John R Hershey and Peder A Olsen. Approximating the Kullback Leibler divergence between Gaussian mixture models. In *2007 IEEE International Conference on Acoustics, Speech and Signal Processing-ICASSP'07*, volume 4, pages IV–317. IEEE, 2007.

[4] Yehor Karpichev, Todd Charter, Jayden Hong, Amir M Soufi Enayati, Homayoun Honari, Mehran Ghafarian Tamizi, and Homayoun Najjaran. Extended reality for enhanced human-robot collaboration: a human-in-the-loop approach. In *2024 33rd IEEE International Conference on Robot and Human Interactive Communication (ROMAN)*, pages 1991–1998. IEEE, 2024.

[5] Lawrence R Rabiner. A tutorial on hidden Markov models and selected applications in speech recognition. *Proceedings of the IEEE*, 77(2):257–286, 1989.

[6] Kjetil Raaen and Ivar Kjellmo. Measuring latency in virtual reality systems. In *Entertainment Computing-ICEC 2015: 14th International Conference, ICEC 2015, Trondheim, Norway, September 29-Ocotober 2, 2015, Proceedings 14*, pages 457–462. Springer, 2015.

[7] Christiane Attig, Nadine Rauh, Thomas Franke, and Josef F Krems. System latency guidelines then and now–is zero latency really considered necessary? In *Engineering Psychology and Cognitive Ergonomics: Cognition and Design: 14th International Conference, EPCE 2017, Held as Part of HCI International 2017, Vancouver, BC, Canada, July 9-14, 2017, Proceedings, Part II 14*, pages 3–14. Springer, 2017.
